# Ketone monoester plus high‐dose glucose supplementation before exercise does not affect immediate post‐exercise erythropoietin concentrations versus glucose alone

**DOI:** 10.14814/phy2.70009

**Published:** 2024-08-22

**Authors:** Emily E. Howard, Jillian T. Allen, Julie L. McNiff, Stephanie D. Small, Kevin S. O'Fallon, Lee M. Margolis

**Affiliations:** ^1^ Military Nutrition Division U.S. Army Research Institute of Environmental Medicine (USARIEM) Natick Massachusetts USA; ^2^ Oak Ridge Institute for Science and Education Belcamp Maryland USA; ^3^ Soldier Sustainment Directorate U.S. Army Combat Capabilities Development Command Soldier Center Natick Massachusetts USA; ^4^ Faculty of Kinesiology & Physical Education University of Toronto Toronto Ontario Canada; ^5^ Soldier Effectiveness Directorate U.S. Army Combat Capabilities Development Command Soldier Center Natick Massachusetts USA

**Keywords:** erythropoiesis, exogenous ketones, ketosis, time trial, β‐Hydroxybutyrate

## Abstract

The objective of this study was to examine the effect of consuming ketone monoester plus a high dose of carbohydrate from glucose (KE + CHO) on the change in erythropoietin (EPO) concentrations during load carriage exercise compared with carbohydrate (CHO) alone. Using a randomized, crossover design, 12 males consumed KE + CHO (573 mg KE/kg body mass, 110 g glucose) or CHO (110 g glucose) 30 min before 4 miles of self‐paced treadmill exercise (KE + CHO:51 ± 13%, CHO: 52 ± 12% V̇O_2peak_) wearing a weighted vest (30% body mass; 25 ± 3 kg). Blood samples for analysis were obtained under resting fasted conditions before (Baseline) consuming the KE + CHO or CHO supplement and immediately after exercise (Post). βHB increased (*p* < 0.05) from Baseline to Post in KE + CHO, with no change in CHO. Glucose and glycerol increased (*p* < 0.05) from Baseline to Post in CHO, with no effect of time in KE + CHO. Insulin and lactate increased (*p* < 0.05) from Baseline to Post independent of treatment. EPO increased (*p* < 0.05) from Baseline to Post in KE + CHO and CHO with no difference between treatments. Although KE + CHO altered βHB, glucose, and glycerol concentrations, results from this study suggest that KE + CHO supplementation before load carriage exercise does not enhance immediate post‐exercise increases in EPO compared with CHO alone.

**New and Noteworthy**

Ketone monoester supplementation during recovery from endurance exercise has been reported to increase blood erythropoietin (EPO) concentrations. Whether EPO concentrations are impacted when a ketone monoester supplement is consumed before an endurance exercise bout is undetermined. Results from Howard et al. show that ketone monoester plus high‐dose glucose supplementation before aerobic exercise does not affect the acute post‐exercise EPO response compared with glucose alone.

## 1 INTRODUCTION

Endurance exercise training can increase maximal oxygen consumption (V̇O_2max_) to improve physical performance (Montero et al., 2015). Increases in V̇O_2max_ result, in part, from improved blood oxygen carrying capacity (Jacobs et al., 2011; Joyner & Coyle, 2008). The hormone erythropoietin (EPO) is central to this response, as increased EPO concentrations stimulate red blood cell production (i.e., erythropoiesis) in bone marrow (Heinicke et al., 2005; Lundby et al., 2007; Montero et al., 2017). The resulting increase in hemoglobin mass allows the cardiovascular system to deliver more oxygen to skeletal muscle during a bout of endurance exercise (Montero et al., 2017), aiding in energy production (e.g., ATP) to sustain physical performance (Jacobs et al., 2011; Joyner & Coyle, 2008). A single bout of prolonged endurance exercise increases circulating EPO concentrations (Schwandt et al., 1991). Interventions that increase circulating EPO concentrations to a greater extent may enhance hematological adaptations to endurance exercise training and further improve physical performance.

Circulating EPO concentrations have been shown to increase following intravenous infusion of the ketone body β‐hydroxybutyrate (βHB) under rested, fasted conditions in healthy, older adults (Lauritsen et al., 2018). Mechanisms underlying the purported relationship between circulating βHB and EPO are not fully understood, and much of the related research has been conducted in disease‐state populations (Cetin et al., 2011; Fernandez‐Pombo et al., 2024; Ferrannini et al., 2017). However, emerging evidence suggests that consuming a ketone monoester (KE) supplement during recovery from endurance exercise and the associated increase in the circulating βHB potentiates the increase in serum EPO that occurs in response to exercise (Evans et al., 2023; Poffé et al., 2023). Evans et al. (2023) reported that consuming 0.29 g KE/kg body mass with carbohydrate and protein after a 60‐min bout of cycling intervals (2‐min 90% VO_2peak_, 2‐min 50% VO_2peak_) resulted in higher EPO concentrations during the initial 4‐h recovery phase compared with carbohydrate and protein supplementation alone. Additionally, Poffé et al. (2023) observed higher circulating EPO concentrations and increased skeletal muscle capillarization during a 3‐week endurance training program when KE was consumed immediately after each training session and 30 min before sleep compared with a medium‐chain triglyceride supplement. While data from these studies are intriguing, it is important to note that these are the only two studies to assess the relationship between KE supplementation and circulating EPO concentrations in response to exercise or exercise training to date. Expanding upon the limited research in this area remains necessary to better understand the impact of KE supplements on circulating EPO concentrations and potential factors that may enhance or negate these effects.

Providing KE in the post‐exercise recovery period contrasts earlier work, which hypothesized that consuming KE before endurance exercise would enhance physical performance by providing an alternative energy substrate that would spare endogenous carbohydrate stores (Cox et al., 2016; Evans et al., 2017). While recent research indicates these effects are unlikely (Howard et al., 2023; Margolis & O'Fallon, 2020), the purported effect of KE consumption on circulating EPO concentrations may represent an alternative ergogenic mechanism for pre‐exercise KE supplementation. The objective of this study was therefore to determine if consuming a KE plus carbohydrate supplement before a 4‐mile bout of load carriage exercise increases EPO concentrations immediately post‐exercise to a greater extent than carbohydrate alone. We hypothesized that consuming a KE plus carbohydrate supplement would result in higher EPO concentrations after a 4‐mile bout of load carriage exercise compared with consuming carbohydrate alone.

## 2 MATERIALS AND METHODS

### 2.1 Participants

Participants in this secondary analysis were part of a larger investigation (www.clinicaltrials.gov as NCT04737694) assessing the impact of ketone monoester plus carbohydrate (KE + CHO) supplementation compared with carbohydrate alone (CHO) on glucose metabolism and physical performance (Howard et al., 2023). Healthy, recreationally active males and females between the ages of 18–39 with a body mass index of less than 30 kg/m^2^ participated in this randomized, double‐blind crossover study. Sixteen males and one female were enrolled to participate. Five were unable to complete both arms of the study due to contracting COVID‐19, knee injury, or no longer wanting to participate. Therefore, data were analyzed and reported on 12 males with complete datasets. Participants provided written informed consent before data collection began. The study was approved by the U.S. Army Medical Research and Development Command Institutional Review Board (Fort Detrick, Fredericksburg, MD, USA: IRB no. M‐10841). Data collection took place at the U.S. Army Research Institute of Environmental Medicine (USARIEM; Natick, MA, USA) between September 2020 and June 2021.

Height (Seritex, Carlstadt, NJ, USA), body mass (WB‐110A, Tanita, Tokyo, Japan), and body composition (dual energy X‐ray absorptiometry, DPX‐IQ, GE Lunar Corporation, Madison, WI, USA) were measured before the start of the study to characterize participants. Peak oxygen uptake (V̇O_2peak_) was also measured at Baseline using a progressive‐intensity treadmill test (4Front, Woodway USA, Inc., Waukesha, WI, USA) and an indirect, open‐circuit respiratory system (True Max 2400, Parvo Medics, Salt Lake City, UT, USA). Participants were 29 ± 5 years and 82 ± 12 kg with an average BMI of 26 ± 3 kg/m^2^, fat mass of 19 ± 6 kg, fat‐free mass of 61 ± 9 kg, and V̇O_2peak_ of 50.5 ± 7.7 mL/kg/min as previously reported (Howard et al., 2023).

### 2.2 Study design

Both study arms began with 72 h of controlled exercise and feeding to ensure glycogen stores were similar between treatments for testing (Margolis et al., 2019). Participants first completed a 50‐min bout of cycle ergometry that consisted of cycling for 2 min at 80 ± 5% V̇O_2peak_ followed by 2 min of recovery at 50 ± 5% V̇O_2peak_ (Howard et al., 2023). A controlled diet was provided for the next 48 h. Participants then reported to the laboratory to complete experiments for the parent study (i.e., 90‐min steady‐state treadmill exercise, time‐to‐exhaustion performance test), which was investigating effects of KE consumption on glucose metabolism and performance. Detailed methods and results for those experiments have been reported previously (Howard et al., 2023). Diets were controlled for the rest of the day after completing testing.

Participants returned to the laboratory the next morning after a 10‐h overnight fast to complete the experiments detailed in this report. They consumed a high‐dose glucose bolus plus ketone monoester (KE + CHO) or glucose alone (CHO) before completing a 4‐mile bout of self‐paced load carriage (LC) treadmill exercise wearing a weighted vest (30% body mass). Participants consumed the same treatment drink (KE + CHO or CHO) before the self‐paced LC as they did the previous day during testing for the parent study. Participants completed the 4‐mile LC bout as rapidly as they could, and performance time data has been reported previously (Howard et al., 2023). Data for this report were derived from analyses of fasted, rested blood samples obtained before (Baseline) participants consumed the KE + CHO or CHO supplement and immediately after (Post) completing exercise. Participants returned to the laboratory to complete the second arm of the study after a minimum 7‐day washout period. Treatment (KE + CHO vs. CHO) order was randomized using a random numbers generator to avoid order bias. Participants and study staff were blinded to the treatment received during each trial.

### 2.3 Controlled diet

Study dietitians prepared and provided all food and beverages except water for the 72‐h controlled feeding period during both treatment arms. Study diets consisted of military combat rations (Meal, Ready‐to‐Eat, Ameriqual, Evansville, IN, USA) and commercially available food items and provided ~6.0 g carbohydrate/kg/d, ~1.2 g protein/kg/d, and 1.0 g fat/kg/d based on Baseline body weight. The same diet was consumed during both treatment arms. Participants returned all wrappers and any uneaten food items to study dietitians to confirm intake and calculate amounts consumed. Energy and macronutrient intake averaged 2890 ± 377 kcal/d, 5.8 ± 0.6 g carbohydrate/kg/d, 1.1 g protein/kg/d, and 1.0 ± 0.1 g fat/kg/d, with no differences between KE + CHO or CHO treatments (Howard et al., 2023).

### 2.4 4‐mile load carriage exercise

Participants arrived to the laboratory after a 10‐h overnight fast. A fasted, rested blood sample was collected upon arrival (Baseline) for blood analyte analysis. Participants then consumed a KE + CHO or CHO study drink and rested for 30 min before starting the 4‐mile LC exercise wearing a weighted vest (30% body mass). This resting period was implemented to allow adequate time to be in ketosis as determined by circulating βHB concentrations (Stubbs et al., 2017). Participants were able to blindly modulate the treadmill speed and were instructed to complete the LC as quickly as possible. The treadmill was set at a constant 1% grade for the entire test (Jones & Doust, 1996). The only feedback provided to participants was the distance covered in half mile increments, at which time heart rate and rate of perceived exertion (RPE) using the Borg 6–20 scale were recorded. A second blood sample was collected immediately after LC (Post). Exercise intensity (%V̇O_2peak_) during the LC was estimated using the American College of Sports Medicine metabolic equation for walking with participant's V̇O_2peak_, LC speed and grade, and body weight plus weighted vest weight (Medicine ACoS, 2021). Participants completed two LC practice sessions before testing to ensure they were familiar with the protocol.

### 2.5 Study supplements

Participants consumed the study drink as an 1100 mL bolus that provided KE + CHO (KE: 573 mg KE/kg body mass + CHO: 110 g glucose) or CHO (110 g glucose) alone. KE was added to the drinks using a commercially available supplement (Pure ΔG® Ketone Ester; H.V.M.N., Miami, FL, USA) containing (R)‐3‐hydroxybutyl (R)‐3‐hydroxybutyrate, water, stevia leaf extract, natural flavors, malic acid, potassium sorbate, and potassium benzoate. The amount of KE provided was based on work from Cox et al. (2016). Study supplements were prepared with matching amounts of carbohydrate and similar color and flavor by the Combat Feeding Division (CFD) within the U.S. Army Combat Capabilities Development Command Soldier Center (Natick, MA, USA). The volume of the KE supplement needed to achieve the desired dose (573 mg KE/kg body mass) was calculated individually for each participant using the concentration reported on the nutrition label (385 mg/mL) and their fasted, rested body weight. Quinine was added to the CHO drink as a flavor match to the bitter taste of the KE, as determined through testing by CFD. Nutrient content was confirmed using gas chromatography (Eurofins Food Integrity and Innovation).

### 2.6 Blood analysis

Blood analytes were analyzed from Baseline and Post blood samples. Circulating βHB concentrations were measured in whole blood by an unblinded staff member (Abbott Precision Xtra Glucose and Ketone Monitoring System; Abbot, Alameda, CA, USA). Serum glucose and glycerol, and plasma lactate and EPO concentrations were determined using enzymatic and colorimetric assays (Beckman Coulter DXC 600 Pro, Beckman Coulter, Brea, CA, USA). Serum insulin concentrations were determined using an advanced automated immunoassay instrument (Immulite 2000: Siemens Healthcare Diagnostic, Deerfield, IL, USA).

### 2.7 Statistical analysis

The statistical power analysis for the current study was conducted using SPSS software (IBM, Armonk, NY, USA). Previously published (Evans et al., 2023) EPO results for KE plus carbohydrate and protein intake compared with carbohydrate and protein intake alone (9.0 ± 2.3 vs. 7.5 ± 1.5 IU/L) using a two‐sided significance level *α* = 0.05, power of 1 – *β* = 0.80, and *r* = 0.8 (hypothesized) were used to determine that a sample size of nine was necessary to achieve statistical significance.

Normality of the data was assessed using Shapiro–Wilk tests for dependent variables. Mixed model ANOVA was used to assess the main effects of treatment, time (or distance), and their interactions for blood analytes and LC pace, heart rate, and RPE. Bonferroni adjustments for multiple comparisons were performed if significant interactions were observed. Average exercise intensity (V̇O_2peak_) during LC and changes in blood analytes (Δ = Post – Baseline) were compared between groups using paired *t*‐tests. Paired *t*‐tests were also used to compare Baseline blood analyte values in KE + CHO versus CHO. Potential effects of trial order (trial 1 vs. trial 2) were assessed using mixed‐model ANOVAs (trial, treatment, trial‐by‐treatment interaction) for all blood analytes. All data are presented as mean ± SD. The α level for significance was set at *p* < 0.05. Data were analyzed using IBM SPSS Statistics for Windows Version 28.0 (IBM Corp. Armonk, NY, USA). Figures were constructed using GraphPad Prism 9 (GraphPad Software, Inc., San Diego, CA, USA).

## 3 RESULTS

Time to complete LC was slower (*p* < 0.05) in KE + CHO (3298 ± 444 sec) compared with CHO (3158 ± 430 sec) as previously reported (Howard et al., 2023). Pace was slower (*p* < 0.001, treatment main effect) throughout LC in KE + CHO compared with CHO regardless of distance completed (Table 1). Heart rate and RPE increased (*p* < 0.001, distance main effect) over the distance completed during LC independent of treatment. Average exercise intensity (%V̇O_2peak_) during the LC exercise was lower (*p* = 0.032) in KE + CHO compared with CHO (51 ± 13% vs. 52 ± 12%).

**TABLE 1:** 4‐mile load carriage exercise.

		Mile				*p* Values		
		1	2	3	4	Distance	Treatment	D × T
Pace (mph)	KE + CHO	4.42 ± 0.57	4.43 ± 0.74	4.42 ± 0.89	4.61 ± 0.98	0.103	<0.001	0.876
	CHO	4.64 ± 0.73	4.65 ± 0.87	4.62 ± 0.77	4.74 ± 0.90			
Heart Rate (BPM)	KE + CHO	148 ± 17^A^	150 ± 15^A^	154 ± 18^B^	157 ± 20^B^	0.003	0.196	0.723
	CHO	151 ± 17^A^	152 ± 17^A^	157 ± 19^B^	163 ± 21^B^			
RPE	KE + CHO	13.0 ± 2.8^A^	14.8 ± 2.0^B^	15.8 ± 1.6^C^	17.1 ± 1.9^D^	<0.001	0.965	0.776
	CHO	12.9 ± 2.7^A^	14.7 ± 1.9^B^	15.6 ± 1.9^C^	17.5 ± 1.8^D^			

*Note*: Values are mean ± SD, *n* = 12. Timepoints not sharing a letter (A, B, C, D) are different; distance main effect, *p* < 0.05. RPE = rate of perceived exertion D × T = distance‐by‐treatment.

**FIGURE 1:**
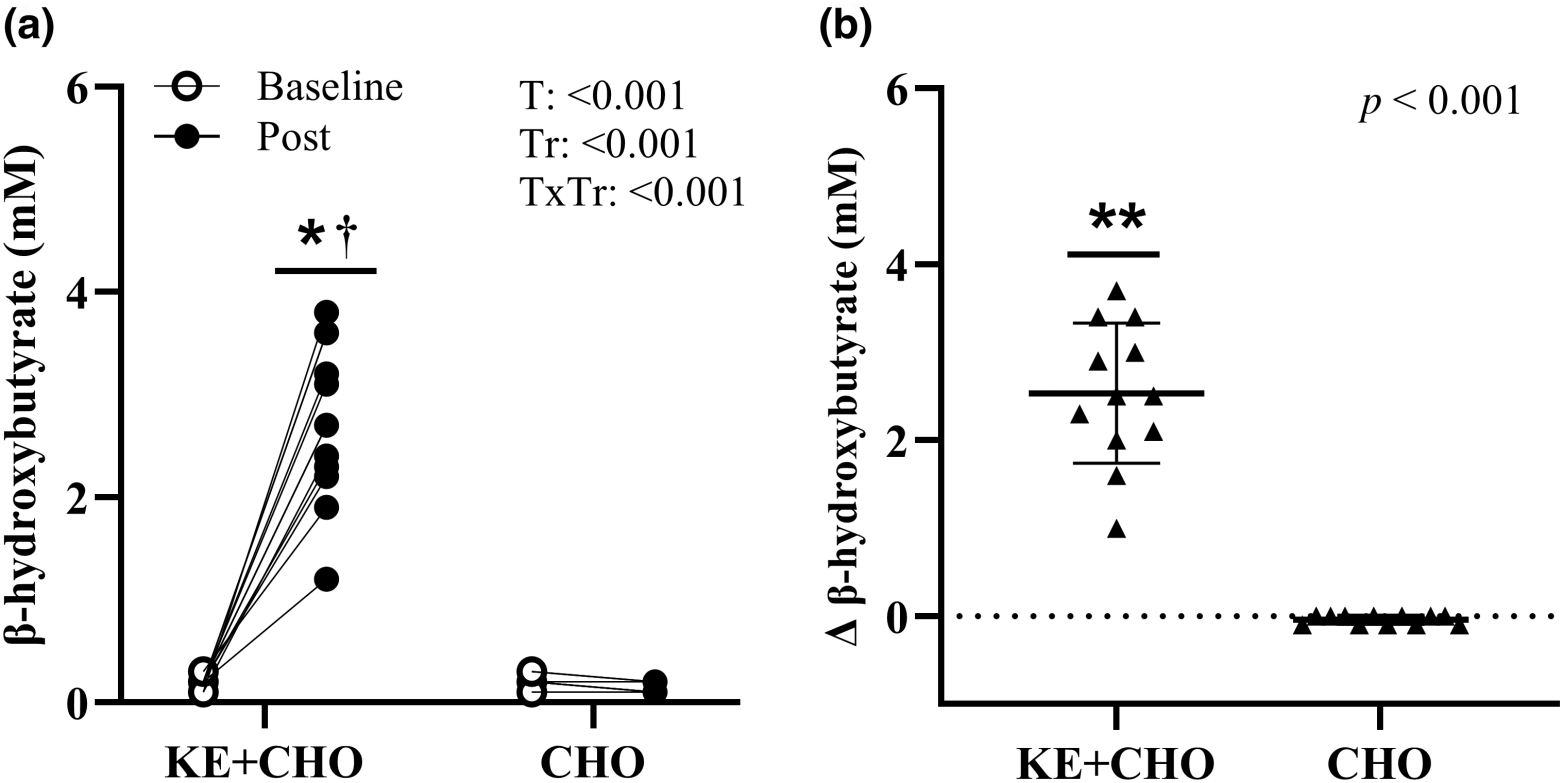
β‐hydroxybutyrate concentrations for volunteers (individual dots) under rested, fasted conditions prior to consuming ketone monoester plus carbohydrate (KE + CHO) or carbohydrate alone (CHO) before exercise (Baseline), and after a 4‐mile bout of self‐paced load carriage treadmill exercise (Post) (a). ∆ = Post – Baseline (b). *n* = 12, *Different than CHO; time‐by‐treatment effect, *p* < 0.05. ^†^Different than Baseline; time‐by‐treatment effect, *p* < 0.05. **Different than CHO, paired *t*‐test, *p* < 0.05. T, time; Tr, treatment.

**FIGURE 2:**
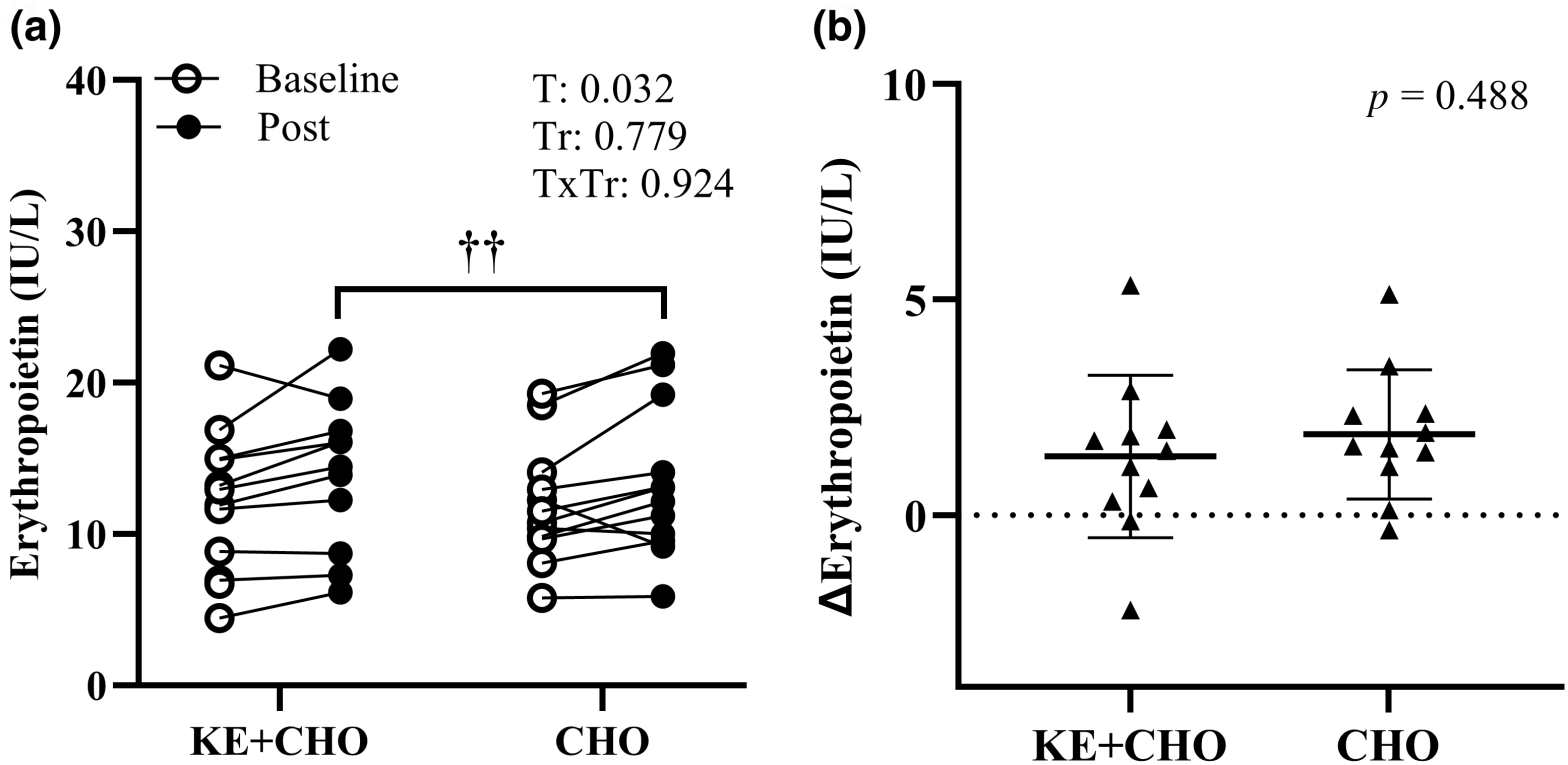
EPO concentrations for volunteers (individual dots) under rested, fasted conditions prior to consuming ketone monoester plus carbohydrate (KE + CHO) or carbohydrate alone (CHO) before exercise (Baseline) and after a 4‐mile bout of self‐paced load carriage treadmill exercise (Post) (a). ∆ = Post – Baseline (b). *n* = 12, ^††^Different than Baseline; time main effect, *p* < 0.05. T, time; Tr, treatment.

**FIGURE 3:**
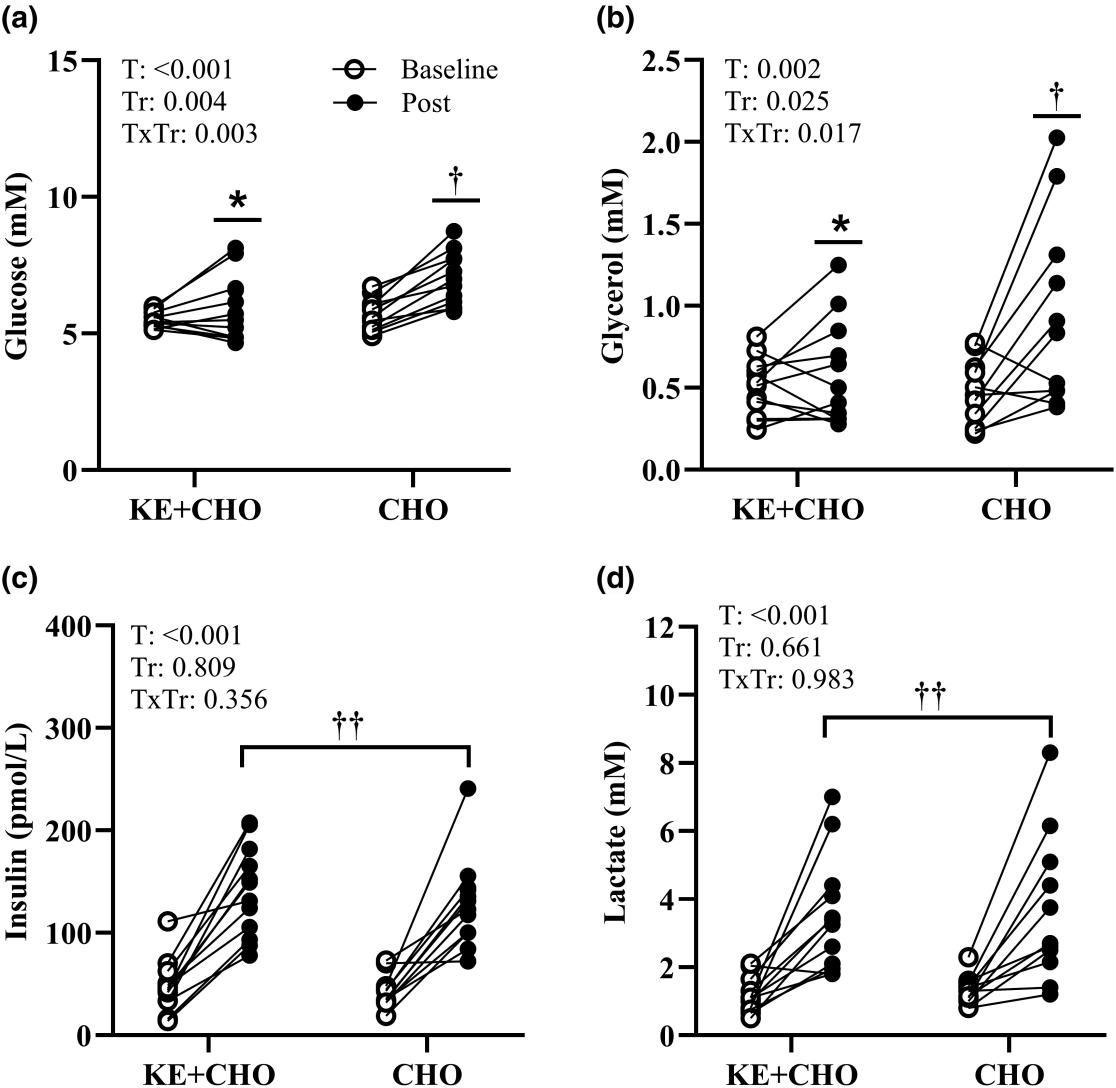
Glucose (a), glycerol (b), insulin (c), and lactate (d) concentrations for volunteers (individual dots) under rested, fasted conditions prior to consuming ketone monoester plus carbohydrate (KE + CHO) or carbohydrate alone (CHO) before exercise (Baseline) and after a 4‐mile bout of load carriage treadmill exercise (Post). *n* = 12, *Different than CHO; time‐by‐treatment effect, *p* < 0.05. ^†^Different than Baseline; time‐by‐treatment effect, *p* < 0.05. ^††^Different than Baseline; time main effect, *p* < 0.05. T, time; Tr, treatment.

βHB increased (*p* < 0.001, time‐by‐treatment effect) from Baseline to Post in KE + CHO, with no change in CHO (Figure 1a,b). βHB was higher (*p* < 0.001, time‐by‐treatment effect) at Post in KE + CHO compared with CHO. EPO increased (*p* = 0.032, time main effect) from Baseline to Post regardless of treatment (Figure 2a,b). Glucose and glycerol increased (*p* < 0.001, time‐by‐treatment effect) from Baseline to Post in CHO, but there was no effect of time in KE + CHO (Figure 3a,b). Glucose and glycerol were lower (*p* < 0.05, time‐by‐treatment effect) at Post in KE + CHO compared with CHO. Insulin and lactate increased (*p* < 0.001, time main effect) from Baseline to Post independent of treatment (Figure 3c,d). Exact values (mean ± SD) and mean differences (95% CI) for blood analytes at Baseline compared with Post are provided for KE+CHO and CHO  (Table 2 **).** Mean differences (95% CI) and effect sizes (95% CI) between deltas (Post minus Baseline) in KE + CHO compared with CHO are also provided (Table 3). There were no differences (*p* > 0.05) in blood analytes at Baseline and no effect (*p* > 0.05) of treatment order (trial 1 vs. trial 2) on blood analyte outcomes (data not shown).

**TABLE 2:** Blood analytes.

	KE + CHO			CHO		
	Baseline	Post	Mean difference (CI)	Baseline	Post	Mean difference (CI)
βHB (mM)	0.19 ± 0.05	2.72 ± 0.77	2.53 (2.21, 2.855)	0.18 ± 0.07	0.14 ± 0.05	−0.04 (−0.36, 0.28)
EPO (IU/L)	12.05 ± 4.75	13.64 ± 4.96	1.59 (−0.39, 3.57)	11.92 ± 3.92	13.38 ± 5.00	1.41 (−0.47, 3.39)
Glucose (mg/dL)	101.58 ± 9.72	106.75 ± 21.56	5.17 (−3.17, 13.5)	101.27 ± 10.78	125.42 ± 17.46	24.15 (15.58, 32.72)
Glycerol (mg/L)	4.68 ± 1.61	5.30 ± 2.94	0.62 (−1.42, 2.67)	4.58 ± 1.83	8.86 ± 5.27	4.28 (2.14, 6.42)
Insulin (μIU/L)	6.84 ± 3.73	20.17 ± 6.42	13.33 (9.35, 17.31)	7.80 ± 6.21	18.54 ± 6.26	10.74 (6.76, 14.72)
Lactate (mM)	1.17 ± 0.52	3.50 ± 1.69	2.33 (1.27, 3.39)	1.33 ± 0.43	3.68 ± 2.17	2.35 (1.24, 3.45)

*Note*: Values are Mean ± SD and mean difference (Post minus Baseline; 95% confidence interval) of the rested, fasted Baseline values before consuming ketone monoester plus carbohydrate (KE + CHO) or carbohydrate (CHO) alone compared with after a 4‐mile bout of self‐paced load carriage treadmill exercise (Post).

**TABLE 3:** Mean difference in blood analytes between treatments.

	Mean difference (CI)	Effect size
βHB (mM)	2.59 (2.08, 3.07)	3.29 (1.81, 4.75)
EPO (IU/L)	−0.55 (−2.11, 1.09)	−0.22 (−0.81, 0.39)
Glucose (mg/dL)	−18.68 (−29.81, −7.55)	−1.13 (−1.88, −0.35)
Glycerol (mg/L)	−3.96 (−6.30, −1.63)	−1.14 (−1.89, −0.36)
Insulin (μIU/L)	2.59 (−2.63, 7.82)	0.32 (−0.27, 0.89)
Lactate (mM)	−0.055 (−1.43, 1.32)	−0.03 (−0.62, 0.57)

*Note*: Values are Mean difference (95% confidence interval) of deltas (Post minus Baseline) between ketone monoester plus carbohydrate (KE + CHO) compared with carbohydrate (CHO) alone. Effect size Cohen's *d*.

## 4 DISCUSSION

The primary finding of this study was that although KE + CHO supplementation before LC increased βHB concentrations to ~3 mM after LC, the acute post‐exercise increase in circulating EPO concentrations remained unchanged compared with consuming CHO alone. Consuming KE + CHO did lower circulating glucose and glycerol after LC compared with CHO alone even though carbohydrate intakes were similar (110 g) between treatments. These results indicate that while KE in combination with a high‐dose glucose bolus impacts circulating substrates, consuming KE + CHO before moderate‐intensity endurance exercise does not enhance post‐exercise increases in EPO concentrations.

KE + CHO supplementation did not affect the acute post‐exercise increase in circulating EPO compared with CHO alone. The lack of an effect of KE + CHO compared with CHO on circulating EPO contradicts previous work reporting higher EPO concentrations when KE was consumed during the recovery phase of endurance exercise (Evans et al., 2023; Poffé et al., 2023) and work showing increases in circulating EPO following intravenous infusion of βHB (Lauritsen et al., 2018). Discordant results between studies likely reflect differences in study design. βHB concentrations are higher following intravenous βHB infusion (~4–5 mmol/L) than KE + CHO supplementation (~3 mmol/L) in the current study. Participants in this study also ingested KE + CHO 30 min before exercise rather than after as shown in previous work (Evans et al., 2023; Poffé et al., 2023). While this noteworthy difference in study design was related to parent study outcomes (Howard et al., 2023), it allowed for the examination of a potential alternative ergogenic mechanism of pre‐exercise KE supplementation on post‐exercise EPO concentrations. The post‐exercise blood draw in this study occurred immediately after exercise and approximately 90 min after consuming the drink. In contrast, Evans et al. (2023) reported differences in EPO concentrations 2, 3, and 4 h after an initial post‐exercise dose of KE. EPO concentrations were also elevated following 4 and 6.5 h of intravenous βHB infusions (Lauritsen et al., 2018). Whether differences in post‐exercise EPO concentrations would have been observed in KE + CHO compared with CHO later during recovery is unclear.

Studies showing an effect of KE supplementation during post‐exercise recovery on circulating EPO concentrations are not without limitations (Evans et al., 2023; Poffé et al., 2023). Evans et al. (2023) reported that KE supplementation coupled with carbohydrate and protein during the initial 4‐h recovery phase of endurance exercise increased EPO concentrations compared with carbohydrate and protein supplementation alone. However, this study was not designed to examine hematological adaptations, and whether the increase in EPO following KE supplementation during endurance exercise recovery alters erythropoiesis is unknown. Work from Poffé et al. (2023) overcomes these limitations, in part, by examining the effects KE supplementation over time. This work showed that KE supplementation 30 min into endurance exercise recovery and again pre‐sleep during 3 weeks of endurance training overload increased EPO concentrations and skeletal muscle capillarization compared with a medium‐chain triglyceride supplement. While it was speculated that increases in EPO with KE supplementation may have contributed to muscular angiogenesis (Poffé et al., 2023), no other hematological (i.e., hemoglobin mass) or aerobic capacity (i.e., VO_2max_) adaptations were examined. Additionally, participants in the KE group had ~20% greater energy intake and ~15% higher training load during the final week of endurance training compared with the control group, making it difficult to isolate the effects of KE alone on EPO concentrations and skeletal muscle capillarization (Hennigar et al., 2020).

The current study showed that post‐LC glucose and glycerol concentrations were lower in KE + CHO compared with CHO. Lower glycerol concentrations in KE + CHO are consistent with the previously reported antilipolytic effect of ketone supplementation (Cox et al., 2016; Poffé et al., 1985), which likely results from ketone bodies exerting negative feedback on their own synthesis (Geisler et al., 2019; Robinson & Williamson, 1980; Taggart et al., 2005). The glucose‐lowering effect of KE supplementation is also well described (Falkenhain et al., 2022). While the exact mechanisms have not been fully elucidated, this may result, in part, from a decrease in hepatic glucose output following KE supplementation (Howard et al., 2023; Mikkelsen et al., 2015; Robinson & Williamson, 1980). It is important to note that the current study design did not allow for measuring blood analytes after consuming the drink and immediately before starting the exercise. This additional timepoint would likely have shown an initial increase in glucose and insulin concentrations that decreased again with exercise, as shown previously with serial blood sampling during steady‐state exercise (Howard et al., 2023).

There are some additional limitations of this work that should be acknowledged. First, the data reported in this manuscript are from a secondary analysis of a study that was not designed to assess markers influencing hematological adaptations. As such, we were unable to report blood markers known to influence iron status such as interleukin‐6 and hepcidin (Peeling et al., 2008, 2009) due to limited archive blood and additional blood sampling timepoints. Measuring changes in these markers along with EPO would provide greater insight into acute alterations in regulators of iron metabolism and erythropoiesis following KE supplementation. Additionally, self‐paced LC performance was faster when consuming CHO compared with KE + CHO, which translated into statistically different estimated exercise intensities between treatments. This was not believed to have impacted the EPO response, however, since the physiological relevance of an approximately 1% difference in exercise intensity is likely limited, and the standardized distance and comparable heart rates between treatments suggest a similar exercise stimulus. Participants were also fasted for study testing. The observed metabolic effects may therefore be different in free‐living scenarios in which individuals consume meals before exercising, especially since feeding can alter absorption and reduce βHB concentrations following KE ingestion (Margolis & O'Fallon, 2020).

In conclusion, consuming KE in combination with a high‐dose glucose bolus 30 min before self‐paced LC elevated βHB to ~3 mM immediately post‐exercise but did not increase EPO concentrations compared with glucose alone. While previous work reported increases in EPO concentrations when KE supplements were consumed during the recovery phase of endurance exercise (Evans et al., 2023; Poffé et al., 2023), results from the current study indicate that consuming KE together with glucose before starting an endurance exercise bout does not enhance the acute post‐exercise EPO response.

## AUTHOR CONTRIBUTIONS

L.M.M. and K.S.O. designed the research; E.E.H., J.T.A., J.L.M, S.D.S., K.S.O., and L.M.M. performed research; E.E.H and L.M.M. analyzed data; E.E.H., K.S.O., and L.M.M. interpreted results; E.E.H. and L.M.M. prepared tables and figures; E.E.H. and L.M.M. drafted the manuscript; and E.E.H., J.T.A., J.L.M., S.D.S., K.S.O., and L.M.M., reviewed and approved the final version.

## FUNDING INFORMATION

This material is based on work supported by the Defense Health Program Joint Program Committee‐5 and appointments to the U.S. Army Research Institute of Environmental Medicine administered by the Oak Ridge Institute for Science and Education (to J.L.M and S.D.S) through an interagency agreement between the U.S. Department of Energy and the U.S. Medical Research and Development Command. The funders of the study had no role in the study design, data collection, data analysis, data interpretation, or writing of the manuscript.

## CONFLICT OF INTEREST STATEMENT

The authors have nothing to disclose.

## ETHICS STATEMENT

The investigators adhered to the policies for protection of human subjects as prescribed in Army Regulation 70–25, and the research was conducted in adherence with the provisions of 32 CFR part 219. The opinions or assertions contained herein are the private views of the authors and are not to be construed as official or as reflecting the views of the Army or the Department of Defense. Any citations of commercial organizations and trade names in this report do not constitute an official Department of the Army endorsement of approval of the products.

## Data Availability

Data is available upon request to the corresponding author.
